# First report of 16SrII–A phytoplasma associated with witches’ broom in *Heliotropium arboreum* (Blanco) Mabb. from Xisha Islands, with *Halticus minutus* as a putative vector

**DOI:** 10.3389/fpls.2025.1681421

**Published:** 2025-09-12

**Authors:** Bao Wang, Likai Feng, Heye Qin, Xuncong Ji, Ruijun Duan, Weikang Huang

**Affiliations:** ^1^ Key Laboratory of Plant Disease and Pest Control of Hainan Province/Institute of Plant Protection, Hainan Academy of Agricultural Sciences (Research Center of Quality Safety and Standards for Agricultural Products of Hainan Academy of Agricultural Sciences), Haikou, China; ^2^ School of Life and Health Science, Hainan University, Haikou, China; ^3^ Institute of Tropical Bioscience and Biotechnology, Chinese Academy of Tropical Agricultural Sciences, Haikou, China

**Keywords:** *Heliotropium arboreum*, 16SrII–A, phytoplasma, molecular identification, *Halticus minutus*

## Abstract

*Heliotropium arboreum* (Blanco) Mabb. (syn. *Tournefortia argentea*), a key pioneer plant in tropical coastal ecosystems, plays a vital role in stabilizing coral islands. In China’s Xisha Islands, *H. arboreum* exhibited witches’ broom symptoms (leaf deformities, reduced leaf size, floral phyllody). PCR assays were employed—using universal and phytoplasma—specific primers to amplify the 16S rRNA, s*ecY*, and *secA* genes—yielding amplicons of 1,248 bp (PV640668), 779 bp (PV854866), and 440 bp (PV854867), confirming a phytoplasma infection (designated *H. arboreum* witches’ broom phytoplasma, HaWB). BLASTn analysis showed 100% sequence identity with 16SrII–group phytoplasmas, while phylogenetic and virtual RFLP analyses classified HaWB as a 16SrII–A subgroup strain. Additionally, morphological and molecular analyses of insects collected from symptomatic plants identified *Halticus minutus* as a putative vector. This is the first report of phytoplasma infecting *H. arboreum* in China, providing critical insights for disease surveillance in fragile coastal ecosystems.

## 1 Introduction

Tree heliotrope (*Heliotropium arboreum* (Blanco) Mabb.), a small tree or shrub in the Boraginaceae family, is a key ecological pioneer species thriving along tropical coasts and islands. It boasts tremendous value in ecosystem restoration, ornamental horticulture, and local sustenance (Cai et al., 2020). Tropical coral island ecosystems (e.g., the Xisha Islands) typically need costly human assistance for recovery after disturbances, primarily because their vegetation structures are relatively simplified and less resilient. As a native species in coral reef ecosystems, remarkably salt– and drought–resistant *H. arboreum* is of paramount importance in stabilizing shorelines and promoting primary succession (Yu, 2021; Manner and Elevitch, 2006). In China, it can be found across Hainan Island, Taiwan Island, as well as the Xisha and Nansha Islands (Cai et al., 2020; Wang et al., 2020). Coral reef islands, isolated from the mainland, are characterized by limited transportation and sparse human activities. Limited studies are reported on diseases and insect pests of *H. arboreum*. Anthropogenic activities serve as one of the primary pathways for long–distance transmission of plant pathogens, as demonstrated by the introduction of pathogens or insect vectors via vehicles, ships, or aircraft (Brown and Hovmøller, 2002; Santini et al., 2018). For example, such activities are among the main causes contributing to the transmission of phytoplasma diseases associated with coconut and cassava (Gurr et al., 2016; Alvarez, 2019). In recent years, with increasing development efforts by the Chinese government in the Xisha Islands, human activities and cargo exchanges have become more frequent, significantly elevating the risk of plant disease and pest spread. Therefore, monitoring plant diseases and insect pests in tropical reef islands is of paramount importance.

Phytoplasmas are cell–wall–less, obligate parasitic prokaryotes residing in plant phloem tissues (Oshima et al., 2004; Kumari et al., 2019; Yu and Wei, 2025). Currently, phytoplasma diseases have been reported in over 100 countries (Kirdat et al., 2023), afflicting more than 1,000 plant species across 98 families and causing characteristic symptoms including witches’ broom, phyllody, stunting, little leaf, and yellowing—losses that are economically significant (McCoy et al., 1989; Bertaccini, 2022). As obligate parasites in plant sieve tubes, phytoplasmas are transmitted by piercing–sucking insects (e.g., leafhoppers, planthoppers) (Wang et al., 2022; Montano et al., 2024). Presently, the taxonomic classification of phytoplasma species primarily relies on conserved gene sequences (e.g., 16S rRNA, *secY*, *secA*, *rp*, *tuf*), which are used to delineate genetic diversity into groups and subgroups (Wei and Zhao, 2022). The phytoplasma group/subgroup classification system is standardized by international taxonomic guidelines, based on the analysis of collective RFLP profiles of the F2n/R2n region of the 16S rRNA gene using 17 restriction endonucleases (Lee et al., 1998; Wang et al., 2024b).

In this study, field surveys were conducted on the Xisha Islands, China, where *H. arboreum* plants were observed to exhibit typical witches’ broom disease symptoms, including shoot proliferation, reduced leaf size, and floral phyllody. Total DNA extracted from diseased leaves was subjected to molecular identification using multiple gene markers. Additionally, a large number of insects belonging to the Miridae family were found around the diseased plants, and *H. minutus* was identified as a putative insect vector for this phytoplasma disease. As a keystone species in tropical coral reef islands, *H. arboreum* plays a critical role in soil stabilization and microhabitat provision, whereby even moderate disease prevalence could compromise ecosystem stability by undermining these functions. Against this backdrop, the study aimed to achieve two primary objectives: (1) to molecularly characterize the phytoplasmas associated with *H. arboreum* witches’ broom disease and determine their taxonomic position within the 16Sr groups; (2) to identify putative insect vectors.

## 2 Materials and methods

### 2.1 Disease incidence survey and sample collection

The survey and sample collection were conducted on March 23, 2025, in the Xisha Islands, China (16.449612°N, 111.508766°E, altitude 8.1 m), with a land area of 0.36 km². A five–point sampling method was adopted to investigate the incidence of witches’ broom disease in *H. arboreum*. At each sampling point, 20 *H. arboreum* plants were inspected, resulting in a total of 100 inspected plants across all five points. Notably, a specific insect was consistently found around diseased plants, hypothesized as a putative vector for disease transmission. From the five survey points, we randomly collected 1 healthy leaf sample (negative control, NC), 3 leaf samples with witches’ broom symptoms (S1, S2, S3). Notably, a specific insect was consistently found around diseased plants, hypothesized as a putative vector for disease transmission. From the five survey points, we randomly collected 1 healthy leaf sample (negative control, NC), 3 suspected diseased samples (S1, S2, S3). Insects were collected using a mouth aspirator: targeting individuals actively foraging or resting on diseased plant tissues (e.g., leaf surfaces, young shoots) and adjacent healthy vegetation (grouped as S1, S2, S3 corresponding to the three diseased plant sample locations). Samples were stored at 4°C during transportation and at −80°C long–term for genomic DNA extraction.

### 2.2 Molecular identification of phytoplasma

One hundred milligrams (100 mg) of young intact leaf samples from *H. arboreum* were weighed; specifically, these samples included the lamina (leaf blade), midribs, and petioles, with no tissue removed. Genomic DNA was extracted using a Plant Genomic DNA Rapid Extraction Kit (Coollaber, Beijing, China) following the manufacturer’s instructions. Total DNA was obtained from one healthy leaf sample and three diseased leaf samples, which were stored at –20°C for subsequent analysis.

For phytoplasma detection, diseased *H. arboreum* leaf samples (S1, S2, S3) and one healthy leaf sample (negative control, NC) were collected. Genomic DNA was extracted, and three target genes were amplified using primer sets with different specificities (sequences in **Table 1**): two genes via a two–step nested PCR strategy (combining universal primers for primary amplification and gene–specific primers for secondary amplification, to improve specificity and sensitivity) and one gene via single–step PCR with gene–specific primers. 16S rRNA gene: nested PCR. Primary reaction with universal primers P1/P7 (Deng and Hiruki, 1991), secondary reaction with R16F2n/R16R2 (Gundersen and Lee, 1996). *secY* gene: nested PCR. Primary reaction with secYwbF1/secYwbR1 (design of this study), secondary reaction with secYwbF2/secYwbR2 (design of this study). *secA* gene: single–step direct amplification from genomic DNA using secAwbF1/secAwbR1 (design of this study). A blank control (BC, ddH_2_O replacing template DNA) was included in all reactions. Each 50 μL PCR reaction mixture contained: 25 μL 2×Es Taq MasterMix, 2 μL each primer (10 μM), 1 μL template DNA (genomic DNA for primary/single–step reactions; 25–fold diluted primary product for secondary reactions), and 20 μL ddH_2_O. Thermal cycling conditions were identical for all reactions: initial denaturation at 95°C for 5 min; 34 cycles of 95°C (30 s), 55°C (30 s), 72°C (1.5 min); final extension at 72°C for 10 min. For the nested PCRs (16S rRNA and *secY* genes), primary PCR products were diluted 25–fold with sterile deionized water and used as templates for secondary PCRs with R16F2n/R16R2 and secYwbF2/secYwbR2, respectively. All PCR products were analyzed by 1% agarose gel electrophoresis, UV–visualized, and photographed.

**Table 1:** Primers used in the study.

Gene	Primer name	Sequence (5’–3’)	Fragment size (bp)	References
16S rRNA	P1	AAGAGTTTGATCCTGGCTCAGGATT	1800	(Deng and Hiruki, 1991)
	P7	CGTCCTTCATCGGCTCTT		
	R16F2n	GAAACGACTGCTAAGACT	1200	(Gundersen and Lee, 1996)
	R16R2	TGACGGGCGGTGTGTACAAACCCCG		
*secY*	secYwbF1	GCATTATCAAGAGATATCTC	1000	this study
	secYwbR1	CAATAGCTACACCAACCAC		
	secYwbF2	TGGAATGAACAAGGTTCTATTGG	800	this study
	secYwbR2	CAGGTAAAGCAGCTAACA		
*secA*	secAwbF1	GACTCGTGAATATGGTTATG	450	this study
	secAwbR1	GATATGTAATCGTCGCAGA		

The target PCR fragments were excised from the agarose gel and purified using the Universal DNA Purification Kit (TIANGEN, Beijing, China) following the manufacturer’s protocol. The ligation reaction (10 μL total volume) contained 1 μL purified PCR product (0.1–0.3 pmol), 1 μL pMD18–T Vector, 3 μL sterile water, and 5 μL Solution I, incubated at 16°C for 30 min. The mixture was then added to 100 μL of *Escherichia coli* DH5α competent cells, incubated on ice for 30 min, heat–shocked at 42°C for 45 s, and chilled on ice for 1 min. Following this, 900 μL of SOC medium was added, and the culture was shaken at 37°C for 1 h. A 200 μL aliquot was plated on LB agar supplemented with ampicillin and incubated overnight at 37°C. Positive clones were verified by colony PCR using the M13F/R primers provided in the kit. To address putative sequence heterogeneity and ensure the reliability of sequencing results, three positive transformants were randomly selected from the validated clones for each sample (including diseased leaf samples S1, S2, S3). These selected clones were sent to Hainan Nanshan Gene Technology Co., Ltd. for Sanger sequencing.

### 2.3 Identification of putative insect vectors

Insects were collected from 3 sampling sites, with 10 individuals per site pooled into one composite sample (3 samples total, each representing a distinct site). The 10 insects in each composite sample were ground together in a mortar with liquid nitrogen, and the powder was collected. Genomic DNA was extracted using the Insect DNA Kit (Omega BIO–TEK, Georgia, USA) following the manufacturer’s instructions. Total DNA was obtained from three insect samples and stored at –20°C for subsequent analysis. Universal primers for phytoplasma (P1/P7 and R16F2n/R16R2) and specific primers (secYwbF1/secYwbR1 and secYwbF2/secYwbR2) were used for two–step amplification of the 16S rRNA and *secY* genes, respectively. PCR amplification protocols, gel electrophoresis, and sequencing steps followed the procedures described in Section 2.2.

Insect morphology was observed, measured, and photographed using a Keyence VHX–7000 digital microscope (Keyence Corporation, Osaka, Japan). Using genomic DNA from the three insect samples as templates, the mitochondrial *COI* gene was amplified by PCR with universal primers LCO1490 (5’–GGTCAACAAATCATAAAGATATTGG–3’) and HCO2198 (5’–TAAACTTCAGGGTGACCAAAAAATCA–3’) (Folmer et al., 1994).

### 2.4 Sequence analysis

The obtained sequences were assembled using SeqMan from the DNAStar software package. The assembled target sequences were subjected to BLASTn searches against the GenBank database, and their similarities were analyzed by comparing with previously reported phytoplasma 16S rRNA, *secY*, and *secA* gene sequences. A phylogenetic tree was constructed via the NJ method in MEGA11, with 1,000 bootstrap replicates (Tamura et al., 2004). Additionally, using 16S rRNA gene sequences, virtual RFLP analysis and similarity coefficient calculations were conducted using the *i*PhyClassifier online tool (https://plantpathology.ba.ars.usda.gov/cgi-bin/resource/iphyclassifier.cgi) to determine phytoplasma taxonomic status (Zhao et al., 2009).

## 3 Result

### 3.1 Symptoms and molecular identification

**Figure 1:**
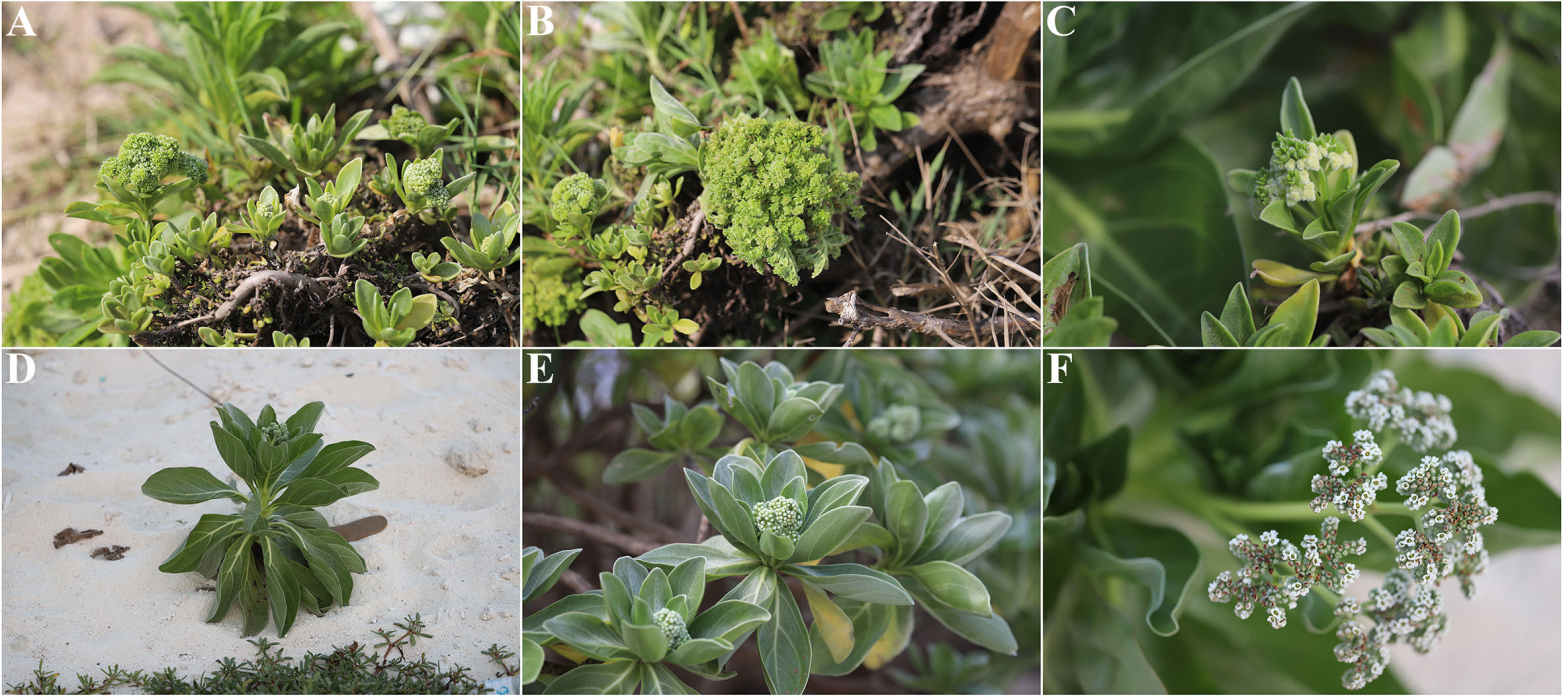
Symptoms of *H*. *arboreum* witches’ broom phytoplasma. **(A–C)** Natural disease manifestations: **(A)** whole–plant witches’ broom characterized by dense shoot proliferation; **(B)** little leaf syndrome with significantly reduced leaf size; **(C)** floral phyllody (abnormal transformation of flowers into leaf–like structures). **(D–F)** Healthy control tissues for comparison: **(D)** intact plant architecture without shoot distortion; **(E)** normal–sized leaves with typical morphology; **(F)** undeformed flowers with functional reproductive structures.

On March 23, 2025, *H. arboreum* plants with witches’ broom symptoms were observed in the Xisha Islands, China. The plants predominantly exhibited symptoms of witches’ broom, little leaf, internode shortening, and phyllody (**Figures 1A–C**), which showed marked differences from healthy plants (**Figures 1D–F**). Using a five–point sampling method, we surveyed 20 plants at each sampling point on the island. The surveys revealed a disease incidence of 5–10% (1–2 diseased plants per sampling point, n=5 points, total 100 plants surveyed), suggesting an early–stage outbreak.

**Figure 2:**
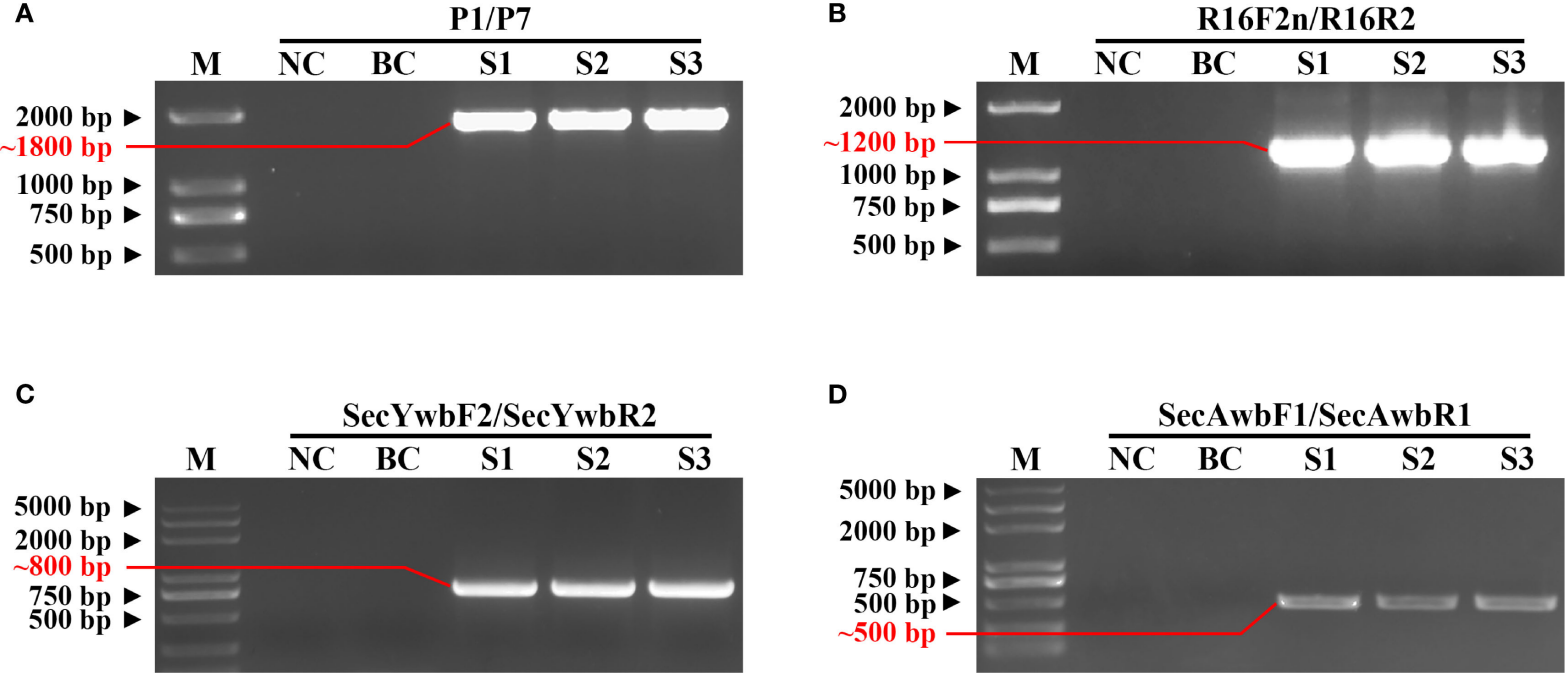
Molecular detection of phytoplasma associated with *H*. *arboreum* witches’ broom disease. **(A)** Agarose gel electrophoresis image of 16S rRNA gene amplification from the pathogen genome using primer pair P1/P7 (~1800 bp product); **(B)** Agarose gel electrophoresis image using primer pair R16F2n/R16R2 (~1200 bp product); **(C)** Agarose gel electrophoresis image using primer pair secYwbF2/secYwbR2 (~800 bp product); **(D)** Agarose gel electrophoresis image using primer pair secAwbF1/secAwbR1 (~500 bp product); M, DNA marker; NC, negative control; BC, blank control; S1–S3, Samples 1–3, respectively.

Multigene PCR detection was conducted to identify phytoplasma in three *H. arboreum* samples with witches’ broom symptoms. For 16S rRNA gene, primary PCR was performed with universal primers P1/P7, and electrophoresis results showed that all three symptomatic samples amplified a ~1800 bp band, while no corresponding band was detected in healthy control samples (**Figure 2A**). Nested PCR (using primers R16F2n/R16R2) yielded a ~1200 bp band in all three symptomatic samples, with no amplification in healthy controls (**Figure 2B**). For the *secY* gene, primary PCR was conducted with specific primers secYwbF1/secYwbR1, but no specific bands were observed by gel electrophoresis. After 25–fold dilution of the primary PCR products with sterile deionized water, nested PCR with primers secYwbF2/secYwbR2 amplified specific ~800 bp bands in the three samples with witches’ broom symptoms (**Figure 2C**). For the *secA* gene, single–step PCR was performed with specific primers secAwbF1/secAwbR1, yielding specific ~500 bp bands in the three symptom–bearing samples (**Figure 2D**). These results confirmed the presence of phytoplasma in the symptomatic samples; the phytoplasma was designated as the Chinese strain of *H. arboreum* witches’ broom phytoplasma.

### 3.2 Identification of putative insect vectors

**Figure 3:**
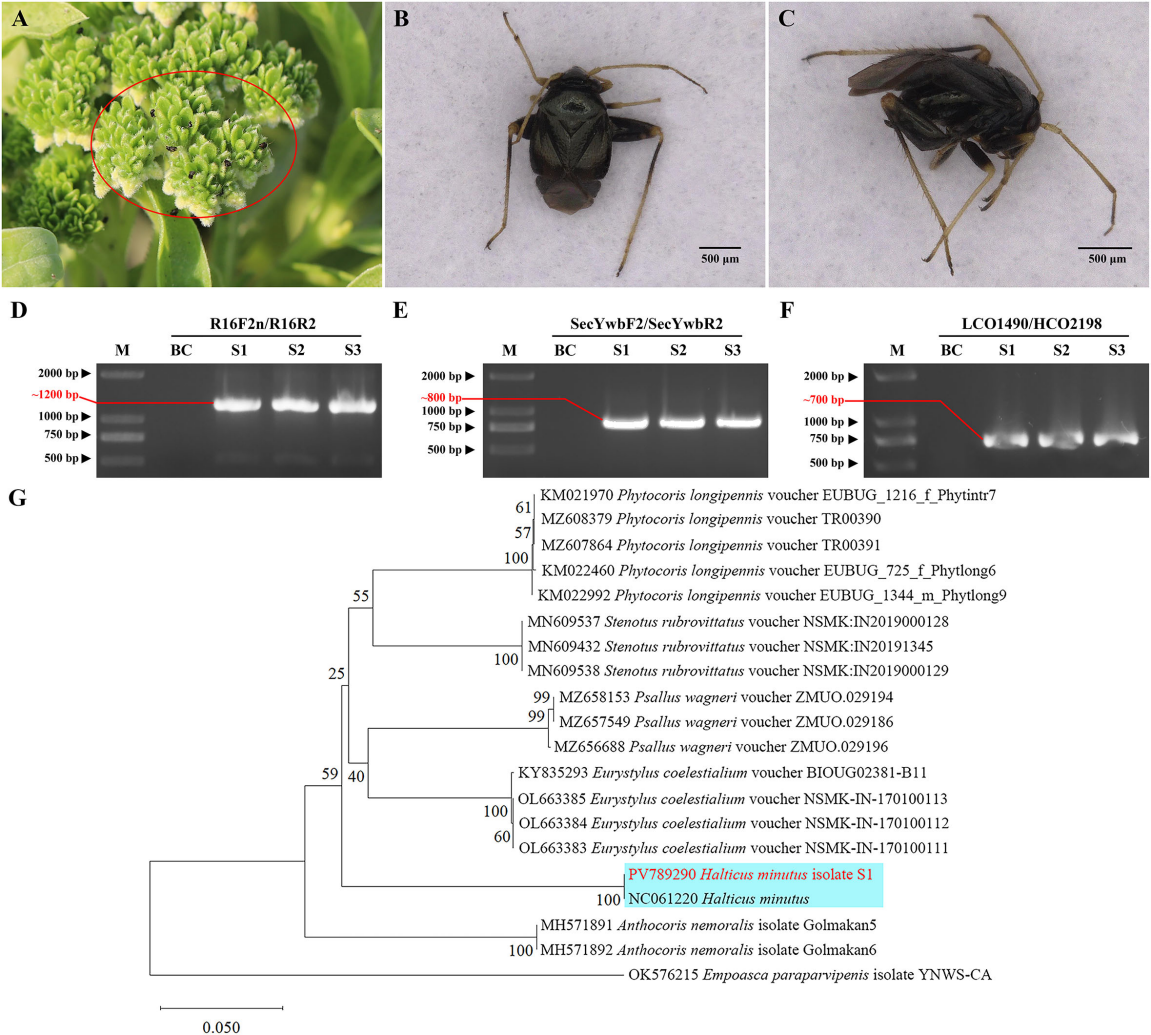
Validation and identification of putative insect vectors transmitting phytoplasma. **(A)** Abundant insects observed around diseased plants (marked by red circles); **(B)** Frontal view of the insect; **(C)** Lateral view of the insect; **(D)** Agarose gel electrophoresis image using primer pair R16F2n/R16R2 (~1200 bp product); **(E)** Agarose gel electrophoresis image using primer pair secYwbF2/secYwbR2 (~800 bp product); **(F)** Agarose gel electrophoresis image using primer pair LCO1490/HCO2198 (~700 bp product). M: DNA marker; BC: blank control; S1–S3: Samples 1–3, respectively. **(G)** Phylogenetic tree of insects based on *COI* gene sequences. A total of 20 representative strains were selected to construct the phylogenetic tree using the Neighbor–Joining method in MEGA11 software, with Bootstrap values set at 1,000. The branch of the *H*. *minutus* is shown with a blue background, and the isolate S1 from this study is highlighted in red bold.

Numerous insects belonging to Heteroptera (suborder Heteroptera, class Insecta) with piercing–sucking mouthparts were observed around plants with witches’ broom symptoms (**Figure 3A**); these Heteropteran insects were considered putative phytoplasma vectors. DNA was extracted from three insect samples (each sample consisted of 10 insects collected around three different symptomatic plants), and nested PCR amplifications of the 16S rRNA and *secY* genes were performed using phytoplasma–specific primers P1/P7, R16F2n/R16R2, secYwbF1/secYwbR1, and secYwbF2/secYwbR2. Gel electrophoresis results showed that phytoplasma 16S rRNA and *secY* genes were present in the three insect DNA samples, with PCR product sizes of approximately 1200 bp and 800 bp, respectively (**Figures 3D, E**), consistent with results from leaf samples of plants with witches’ broom symptoms. Sequencing revealed 100% gene sequence identity between phytoplasma strains from leaves of symptomatic plants and insects, confirming the presence of the same phytoplasma.

The adults measured approximately 2.0 mm in length, were black and glossy, with slender yellowish–brown antennae. The pronotum was short and broad, with straight anterior and lateral margins, and a posterior margin projecting backward in an arc. The scutellum was triangular, the forewing corion was short, broad, and dark brown, the legs were yellowish–brown to dark brown, the hind femora were short but stout, the abdomen was black with hairs, and the mouthparts were piercing–sucking (**Figures 3B, C**)—morphological traits fully consistent with the description of *Halticus minutus* provided by Wang et al. (2014). Additionally, to confirm the insect’s taxonomic identity molecularly, the mitochondrial cytochrome c oxidase subunit I (*COI*) gene was amplified from insect DNA using primers LCO1490/HCO2198. Gel electrophoresis showed ~700 bp PCR products for all three insect samples, with no bands in the blank control (**Figure 3F**). Sequencing revealed 100% sequence identity among the three insect samples, indicating genetic homogeneity of the tested population. The *COI* gene sequence of sample S1 was submitted to the NCBI GenBank database under accession number PV789290. BLAST analysis showed 99.85% identity to the *COI* gene fragment of *H. minutus* mitochondrion genome (NC061220) (coverage=100%, e–value=0), 83.53% identity with *Eurystylus coelestialium* voucher NSMK–IN–170100111 *COI* gene (OL663383), and 82.95% identity with *Psallus wagneri* voucher ZMUO.029196 *COI* gene (MZ656688). Notably, all three reference species belong to Hemiptera: Miridae, confirming the insect’s placement in the family Miridae; the substantially higher identity to *H. minutus* further narrows its taxonomic status to the genus *Halticus*. To further verify, a phylogenetic tree was constructed based on the *COI* gene sequences using MEGA11 software. The ingroup included our S1 sequence, 15 reference *COI* sequences of Miridae species, and 2 reference *COI* sequences of Anthocoridae, with *Empoasca paraparvipenis* (OK576215) as outgroup. The result shows that isolate S1 clustered with *H. minutus* (**Figure 3G**). Thus, combining morphological congruence with *H. minutus* (Wang et al., 2014) and molecular evidence, the insect was tentatively identified as *Halticus minutus*.

### 3.3 BLASTn analysis of multigene sequences

To characterize the phytoplasma associated with *H. arboreum* witches’ broom (HaWB), three conserved genes (16S rRNA, *secY*, and *secA*) were amplified using phytoplasma–universal primers for 16S rRNA (R16F2n/R16R2) and phytoplasma–specific primers for *secY* (secYwbF2/secYwbR2) and *secA* (secAwbF1/secAwbR1), yielding amplicons of ~1,248 bp, ~779 bp, and ~440 bp, respectively. Sequencing of the three gene fragments from three independent samples revealed 100% sequence identity within each gene across samples, indicating genetic homogeneity of the HaWB phytoplasma population. Consensus sequences were submitted to the NCBI GenBank database under accession numbers PV640668 (16S rRNA), PV854866 (*secY*), and PV854867 (*secA*). BLASTn analysis of these sequences consistently showed 100% identity between the HaWB strain and multiple phytoplasma strains in the 16SrII group, including *Crotalaria* witches’ broom (CrWB–Hnsy1, EU650181; CrWB–Hnsy2004, JF834194), sweet potato witches’ broom (SPWB, CP171825), *Vigna unguiculata* witches’ broom (HNNKY–3, OR666421; HNNKY–3, OR661282), *Vigna angularis* witches’–broom (Ab_1, PQ619118), and *Tephrosia purpurea* witches’ broom (TpWB–hnld, MW603929). Collectively, the congruent 100% sequence identity across 16S rRNA, *secY*, and *secA* genes conclusively classified the HaWB phytoplasma within the 16SrII group.

### 3.4 Phylogenetic tree and virtual RFLP analysis

**Figure 4:**
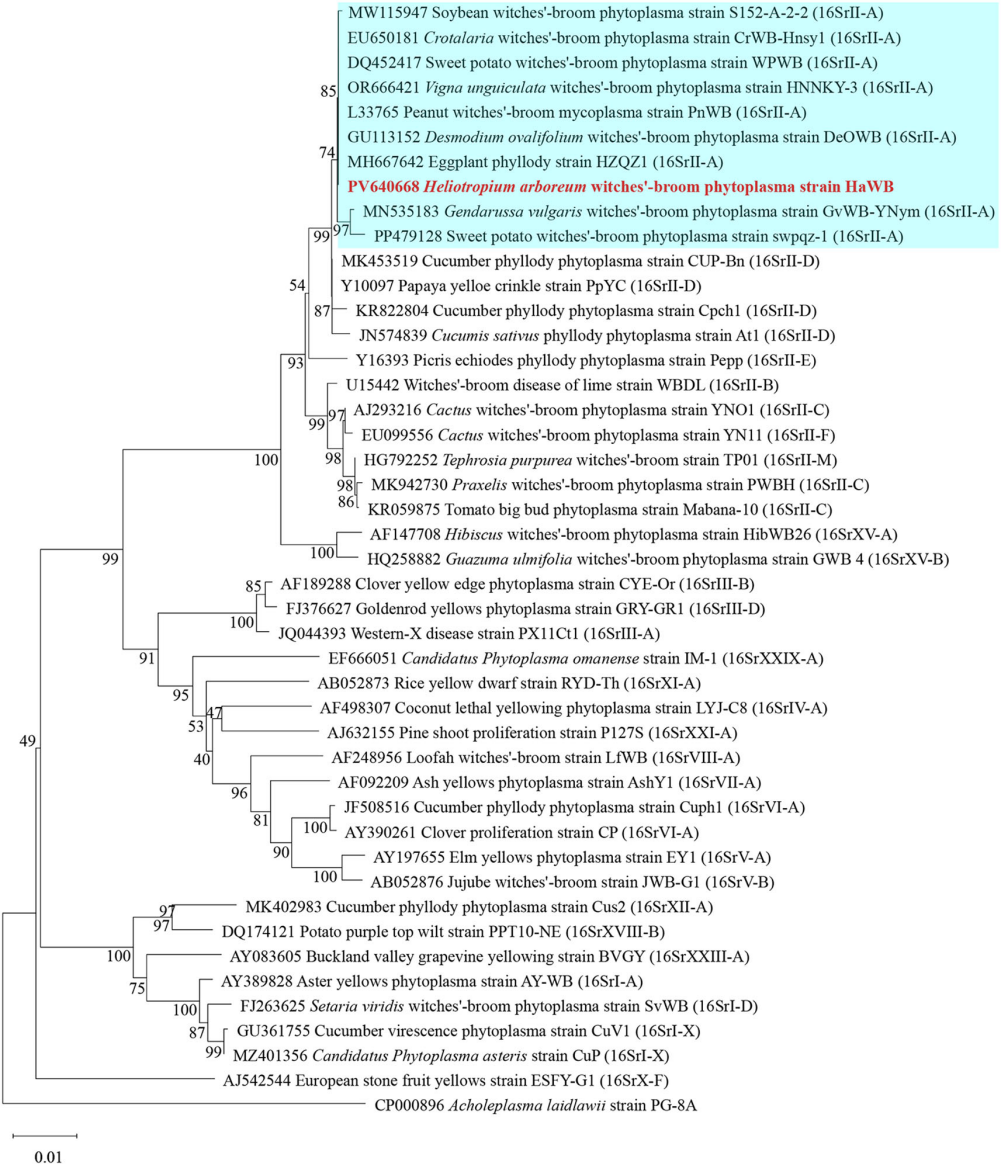
Phylogenetic tree of *H. arboreum* witches’ broom phytoplasma based on 16S rRNA gene sequences. A total of 44 representative strains were selected to construct the phylogenetic tree using the Neighbor–Joining method in MEGA11 software, with Bootstrap values set at 1,000. The branch of the 16SrII–A subgroup is shown with a blue background, and the strain HaWB from this study is highlighted in red bold.

Phylogenetic analysis based on the 16S rRNA gene sequences was performed using the Neighbor–Joining method with 1000 bootstrap replicates in MEGA software. The results showed that phytoplasma strain HaWB, causing witches’ broom disease in *H. arboreum*, clustered with 16SrII–A subgroup members, including *Crotalaria* witches’ broom phytoplasma (EU650181), Sweet potato witches’ broom phytoplasma (DQ452417), and *Vigna unguiculata* witches’ broom phytoplasma (OR666421), within a single evolutionary clade (**Figure 4**). Strains used for the analysis, along with their associated plant diseases, geographical locations, phytoplasma subgroup classifications, and GenBank Accession numbers, are listed in **Supplementary Table S1**.

**Figure 5:**
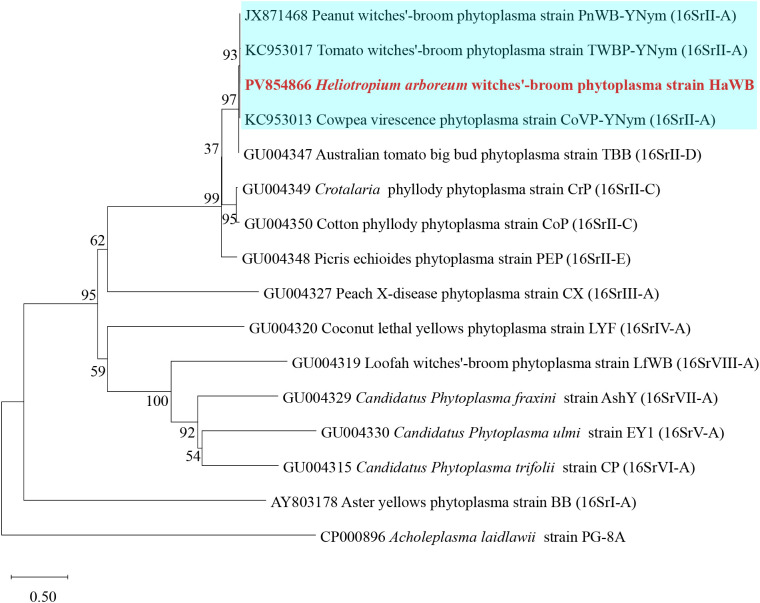
Phylogenetic tree of *H. arboreum* witches’ broom phytoplasma based on *secY* gene sequences. A total of 15 representative strains were selected to construct the phylogenetic tree based on *secY* gene. Using the Neighbor–Joining method in MEGA11 software, with Bootstrap values set at 1,000. The branch of the 16SrII–A subgroup is shown with a blue background, and the strain HaWB from this study is highlighted in red bold.

Phylogenetic analysis based on the *secY* gene sequences was performed using the Neighbor–Joining method with 1000 bootstrap replicates in MEGA software. The results showed that phytoplasma strain HaWB, causing witches’ broom disease in *H. arboreum*, clustered with 16SrII–A subgroup members, including Peanut witches’–broom phytoplasma (JX871468), Tomato witches’–broom phytoplasma (KC953017), and Cowpea virescence phytoplasma (KC953013), within a single evolutionary clade (**Figure 5**). Strains used for the analysis, along with their associated plant diseases, geographical locations, phytoplasma subgroup classifications, and GenBank Accession numbers, are listed in **Supplementary Table S2**.

**Figure 6:**
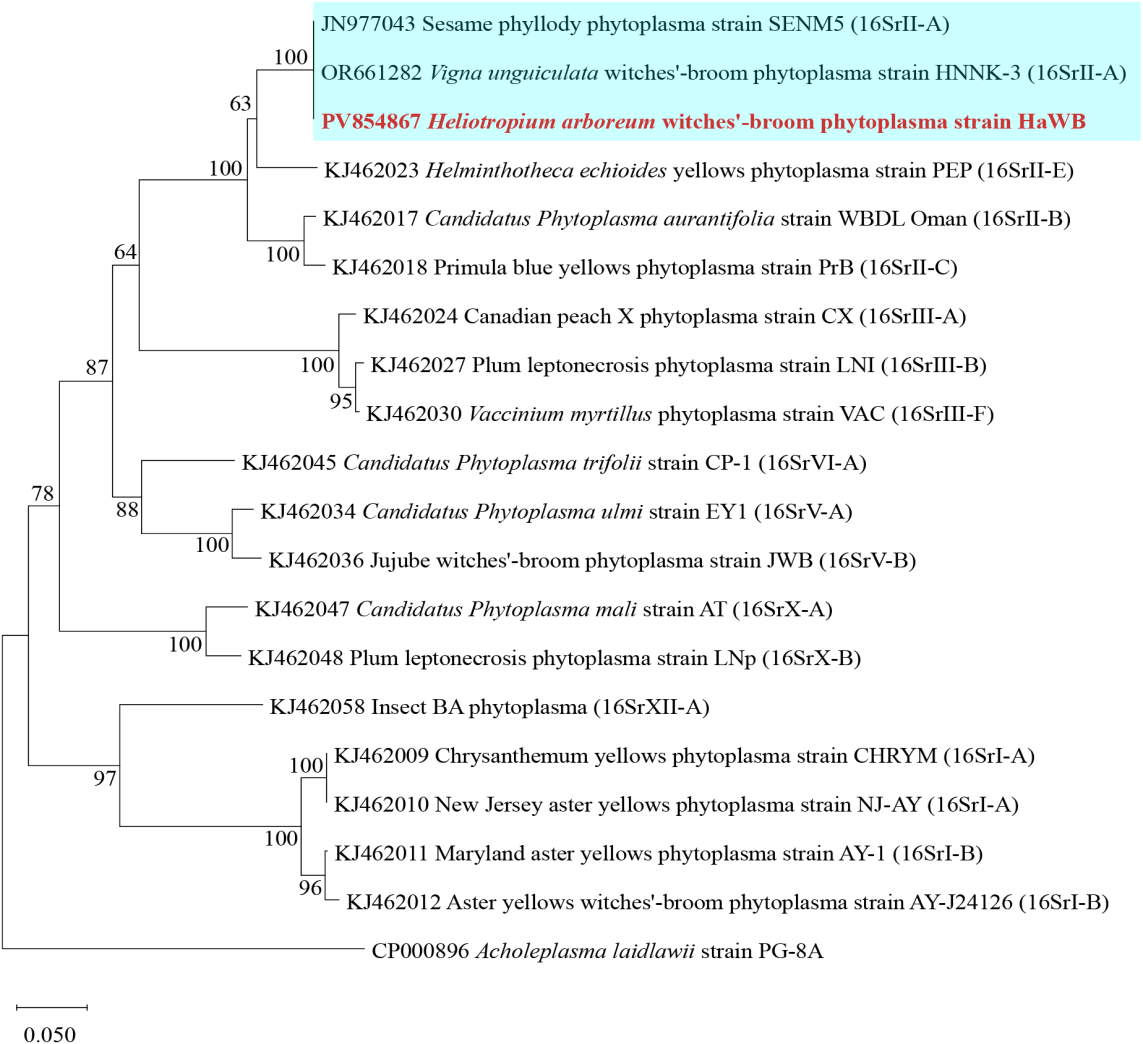
Phylogenetic tree of *H. arboreum* witches’ broom phytoplasma based on *secA* gene sequences. A total of 19 representative strains were selected to construct the phylogenetic tree based on secA gene. Using the Neighbor–Joining method in MEGA11 software, with Bootstrap values set at 1,000. The branch of the 16SrII–A subgroup is shown with a blue background, and the strain HaWB from this study is highlighted in red bold.

Phylogenetic analysis based on the *secA* gene sequences was performed using the Neighbor–Joining method with 1000 bootstrap replicates in MEGA software. The results showed that phytoplasma strain HaWB, causing witches’ broom disease in *H. arboreum*, clustered with 16SrII–A subgroup members, including Sesame phyllody phytoplasma (JN977043), and *Vigna unguiculata* witches’ broom phytoplasma (OR661282), within a single evolutionary clade (**Figure 6**). Strains used for the analysis, along with their associated plant diseases, geographical locations, phytoplasma subgroup classifications, and GenBank Accession numbers, are listed in **Supplementary Table S3**.

To further clarify its taxonomic classification, the 16S rRNA gene sequence of *H. arboreum* phytoplasma was analyzed via virtual RFLP using the *i*PhyClassifier online tool. Results showed that the virtual RFLP profile of strain HaWB—responsible for witches’ broom, reduced leaf size, and phyllody in *H. arboreum*—exhibited a perfect similarity coefficient (1.00) to the reference strain of the 16SrII–A group, Peanut witches’ broom phytoplasma (L33765). Notably, the virtual restriction profiles of all 17 tested enzymes were completely identical providing definitive evidence that the *H. arboreum* witches’ broom phytoplasma strain HaWB belongs to the 16SrII–A subgroup.

## 4 Discussion

Phytoplasmas are important prokaryotic pathogens causing diverse plant diseases globally. In China, over 100 phytoplasma–associated diseases have been documented, primarily linked to 11 16Sr groups (16SrI to 16SrXIV) with distinct geographical and host specificities (Wang et al., 2022; Li et al., 2025). This study provides the first molecular evidence of a 16SrII–A subgroup phytoplasma (designated HaWB) infecting *H. arboreum*—a keystone pioneer species in coral island ecosystems—on China’s ecologically fragile Xisha Islands. Phylogenetic and multigene analyses (16S rRNA, *secY*, *secA*) confirmed HaWB as a 16SrII–A member, a subgroup known to infect economically critical crops such as sweet potato (Li et al., 2025), peanut (Li et al., 2014), *Vigna unguiculata* (Wang et al., 2024a), *Cajanus scarabaeoides* (Wang et al., 2018), and areca nut (Lin et al., 2023). Notably, HaWB shared 100% sequence identity across all three genes with sweet potato witches’–broom phytoplasma (CP171825), suggesting putative cross–ecosystem transmission (from agricultural systems to coral island habitats) via polyphagous vectors—with significant ecological implications given *H. arboreum*’s role in shoreline stabilization.

Field surveys using a five–point sampling method revealed a 5–10% disease incidence in *H. arboreum*, though this may underestimate true prevalence due to asymptomatic infections common in early phytoplasma outbreaks. Concurrently, numerous Miridae insects were observed on diseased plants; their morphology (e.g., 2.0 mm length, black glossy cuticle, piercing–sucking mouthparts) matched *H. minutus* (Wang et al., 2014), and mitochondrial *COI* gene analysis confirmed 99.85% identity with *H. minutus* (NC061220). As a polyphagous pest of crops including sweet potato, soybean, and cowpea (Wang et al., 2014; Tong and Wang, 1987), its host range overlaps strongly with 16SrII–A phytoplasma–susceptible plants, supporting vector putative. Previous studies have documented associations between mirid insects and phytoplasmas (Mitchell, 2004), including *Lygus rugulipennis* with tomato stolbur and *H. minutus* with sweet potato little leaf; however, Wheeler (2001) emphasized that all such associative records lack confirmation of vector capacity and require further experimental validation. Notably, to date, no controlled transmission experiments (e.g., cage–based acquisition–inoculation trials) have been conducted for *H. minutus* or any other species within the genus *Halticus*; thus, this represents the most urgent task for future research.

Two methodological considerations arise. First, transmission assays were unfeasible as field–collected *H. minutus* died during transport from the remote islands. However, indirect evidence supports its role: (i) consistent association with symptomatic *H. arboreum*; (ii) 100% identity between phytoplasma genes (16S rRNA, *secY*) from insects and plants; (iii) overlapping host ranges. Future studies should prioritize on–site trials or laboratory colonization, leveraging island research stations to overcome logistical barriers. Second, while the 3 diseased samples may seem limited, the 0.36 km² island’s homogeneous *H. arboreum* distribution (a trait of isolated reef ecosystems with simplified vegetation) justifies this. The five–point sampling captured distinct microhabitats (e.g., coastal vs. inland), and identical 16S rRNA, *secY*, and *secA* sequences across samples confirmed a single HaWB strain—consistent with low phytoplasma divergence in small, closed ecosystems. For such systems, representativeness depends on microhabitat coverage over quantity.

Global records from the GBIF database (https://www.gbif.org/species/5812900) indicate *H. minutus* has a widespread distribution (e.g., China, Australia, the Americas), but its presence on the Xisha Islands—over 300 km from the nearest mainland—raises questions about dispersal. We hypothesize two pathways: (1) passive aerial dispersal facilitated by its small size (~2.0 mm adults); and (2) anthropogenic introduction via cargo (e.g., sweet potato, peanut) linked to increased regional development (Santini et al., 2018). This highlights the need for enhanced surveillance of both *H. minutus* and phytoplasma diseases in other reef island plants, as even low incidence (5–10%) in a foundational species like *H. arboreum* could disrupt coral island stabilization and primary succession.

Overall, this study expands the known host range of 16SrII–A phytoplasmas to a key coastal pioneer species and identifies a candidate vector in a vulnerable ecosystem. It underscores the urgency of strengthening phytosanitary measures to prevent disease spread, particularly in isolated island systems where ecological recovery from disturbances is inherently limited.

## 5 Conclusion

This study characterized the phytoplasma strain HaWB isolated from *H. arboreum*. Multigene sequence analysis (including 16S rRNA, *secY*, and *secA* genes), phylogenetic analysis, and RFLP analysis confirmed that the *H. arboreum* witches’ broom phytoplasma strain HaWB belongs to the 16SrII–A subgroup. Additionally, *H. minutus* may be one of the insect vectors transmitting this phytoplasma. In summary, to the best of our knowledge, this study reports for the first time the occurrence of *H. arboreum* witches’ broom disease in the Xisha Islands, China, providing a scientific basis for the prevention and control of this disease.

## Data Availability

The datasets presented in this study can be found in online repositories. The names of the repository/repositories and accession number(s) can be found in the article/**Supplementary Material**.
