# Supplementary figures and images for: First report of 16SrII–A phytoplasma associated with witches’ broom in Heliotropium arboreum (Blanco) Mabb. from Xisha Islands, with Halticus minutus as a putative vector
Source: Front Plant Sci. 2025 Sep 12;16:1681421. doi: 10.3389/fpls.2025.1681421 (PMC12463901; doi:10.3389/fpls.2025.1681421)

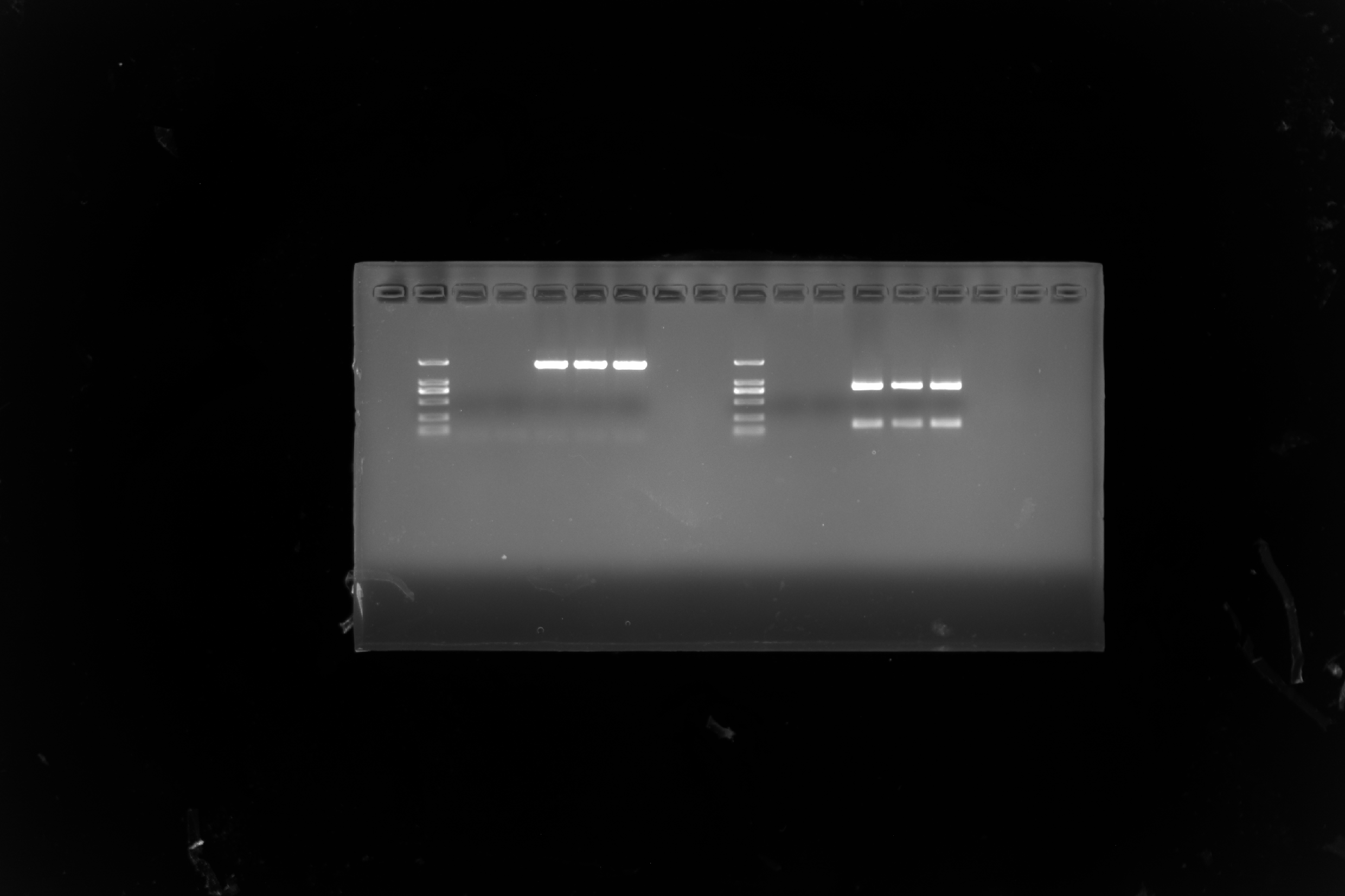

Supplement: Supplementary file 1 [file DataSheet1.zip › Supplementary Gel Images-1681421/FIG2A.tif]

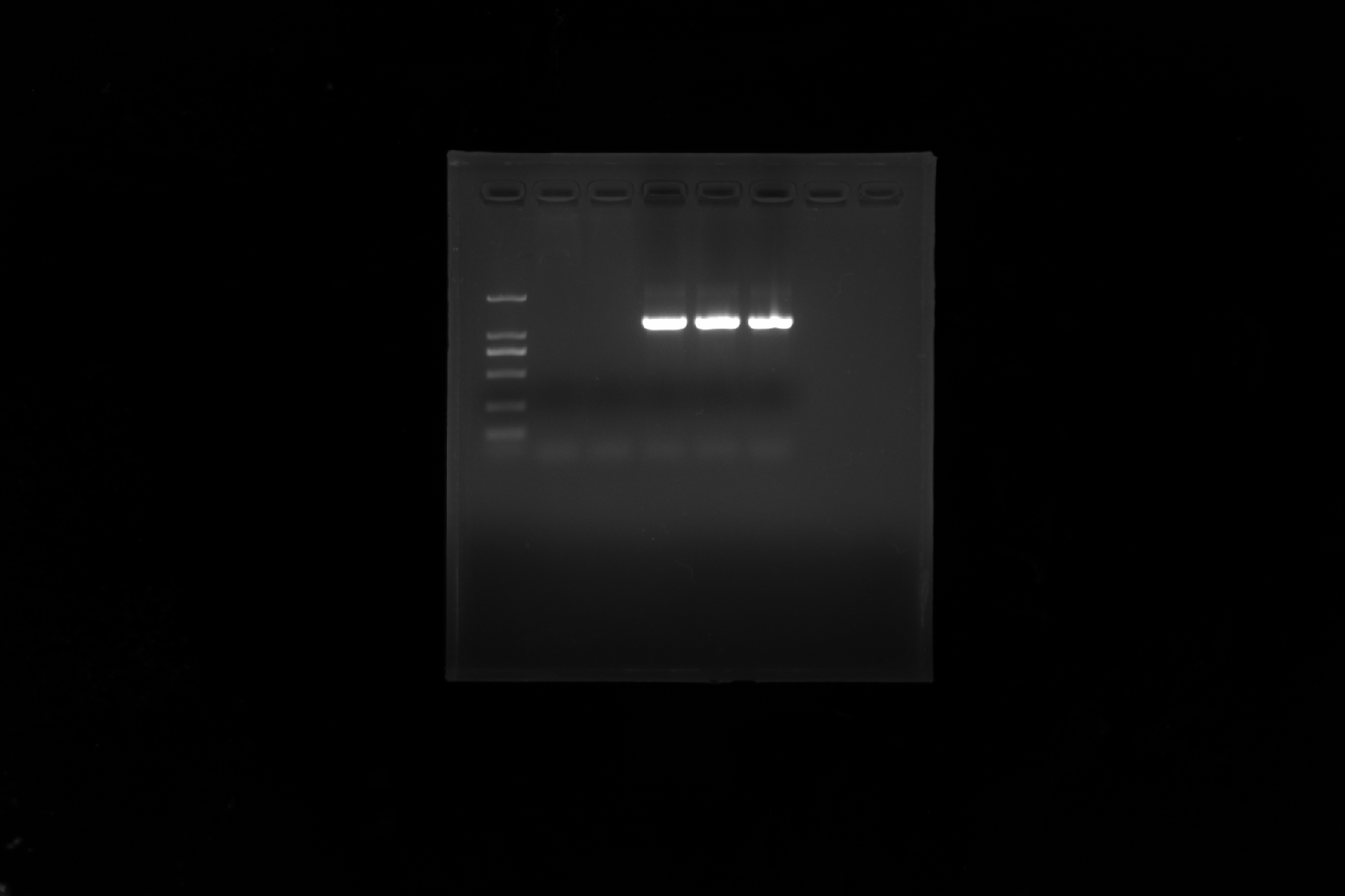

Supplement: Supplementary file 1 [file DataSheet1.zip › Supplementary Gel Images-1681421/FIG2B.tif]

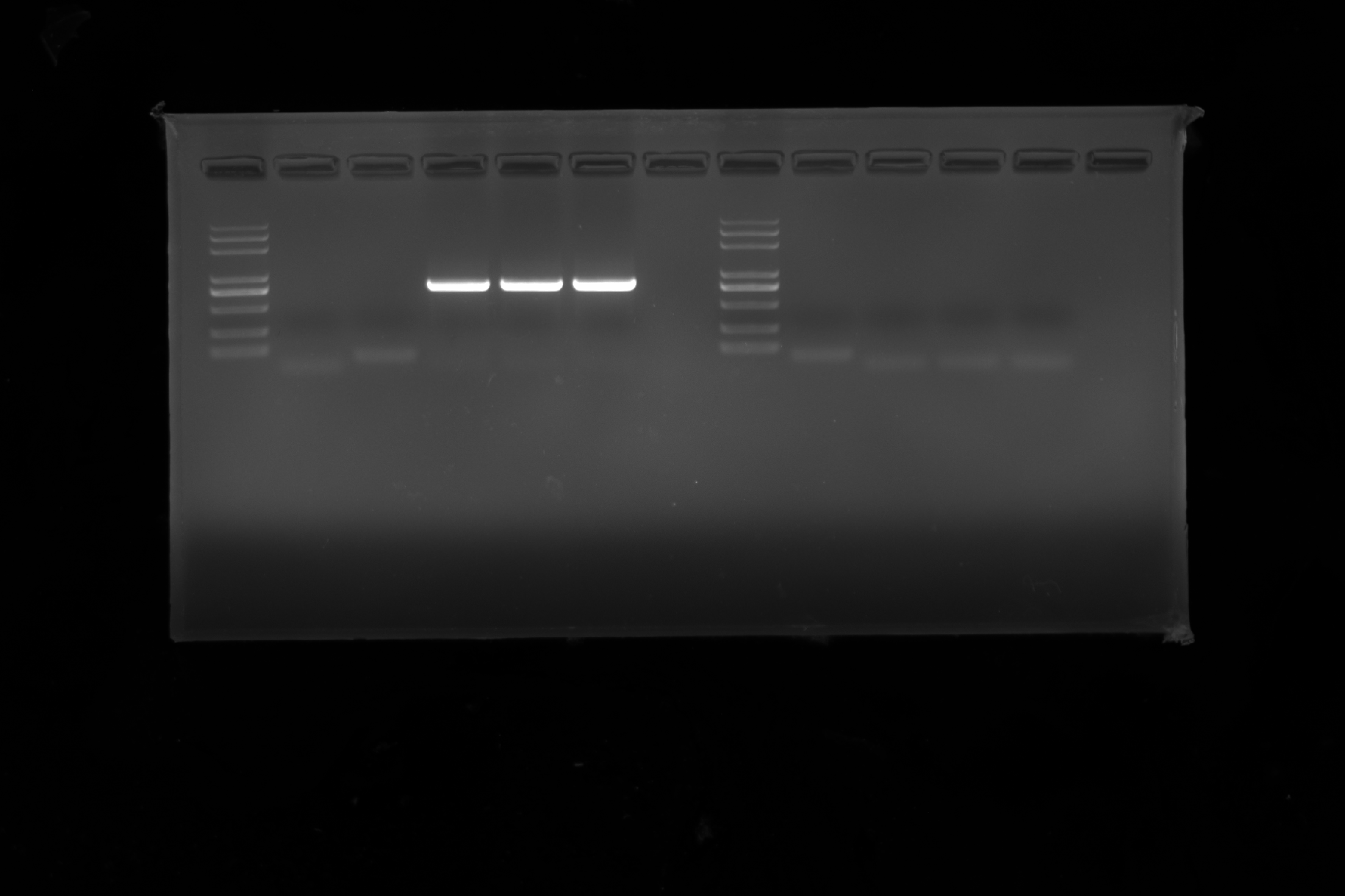

Supplement: Supplementary file 1 [file DataSheet1.zip › Supplementary Gel Images-1681421/FIG2C.tif]

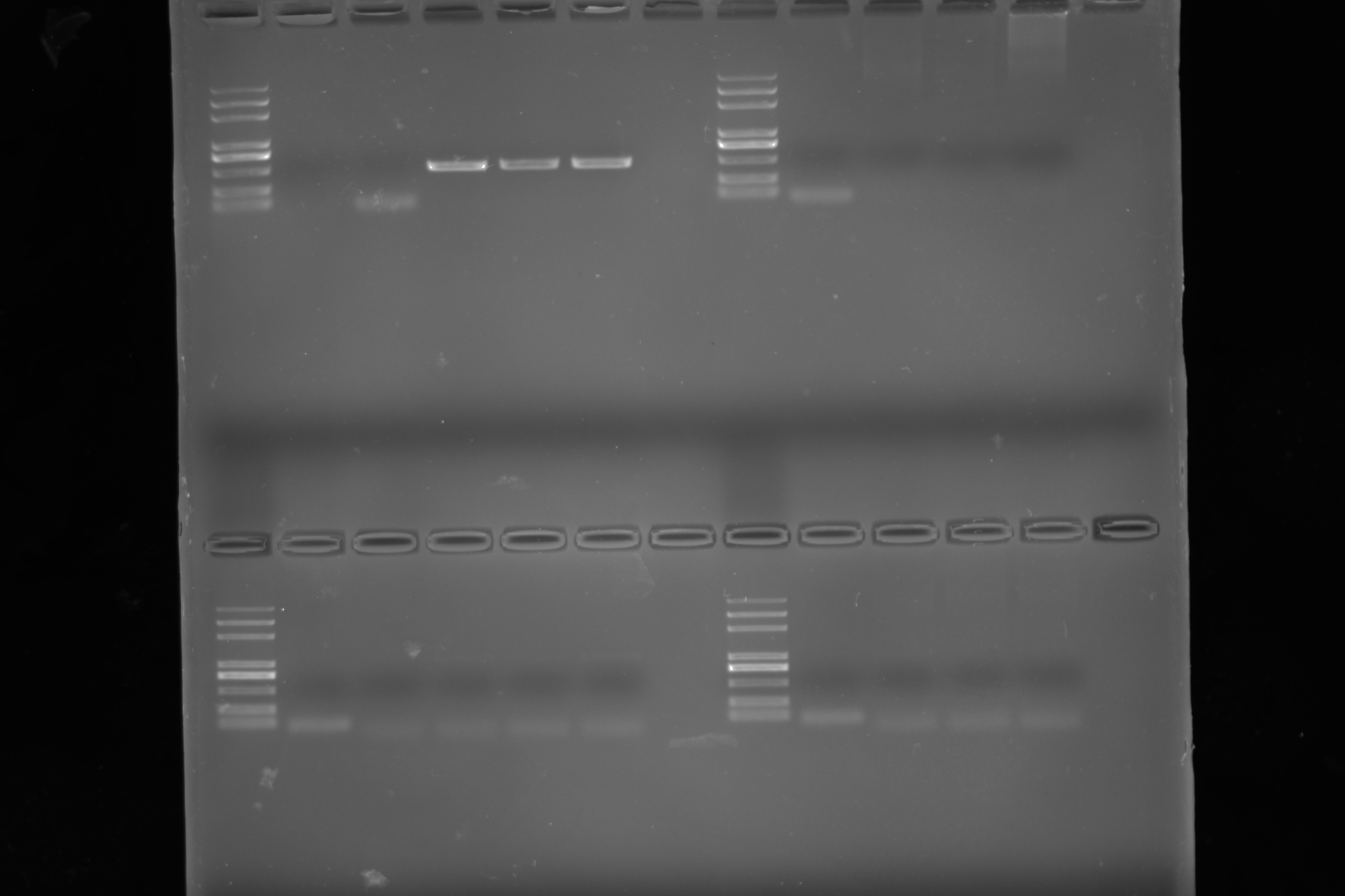

Supplement: Supplementary file 1 [file DataSheet1.zip › Supplementary Gel Images-1681421/FIG2D.tif]

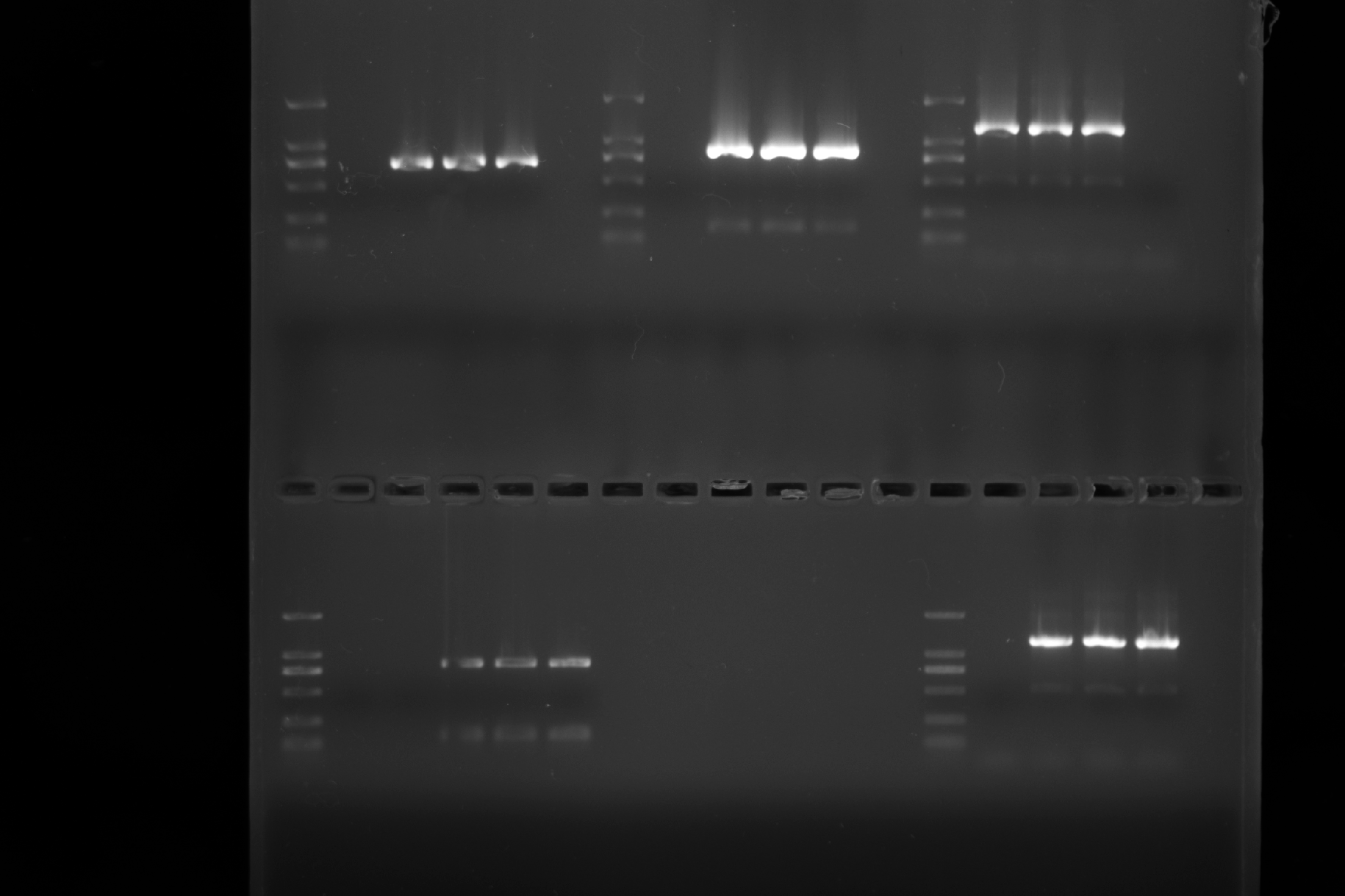

Supplement: Supplementary file 1 [file DataSheet1.zip › Supplementary Gel Images-1681421/FIG3D and F.tif]

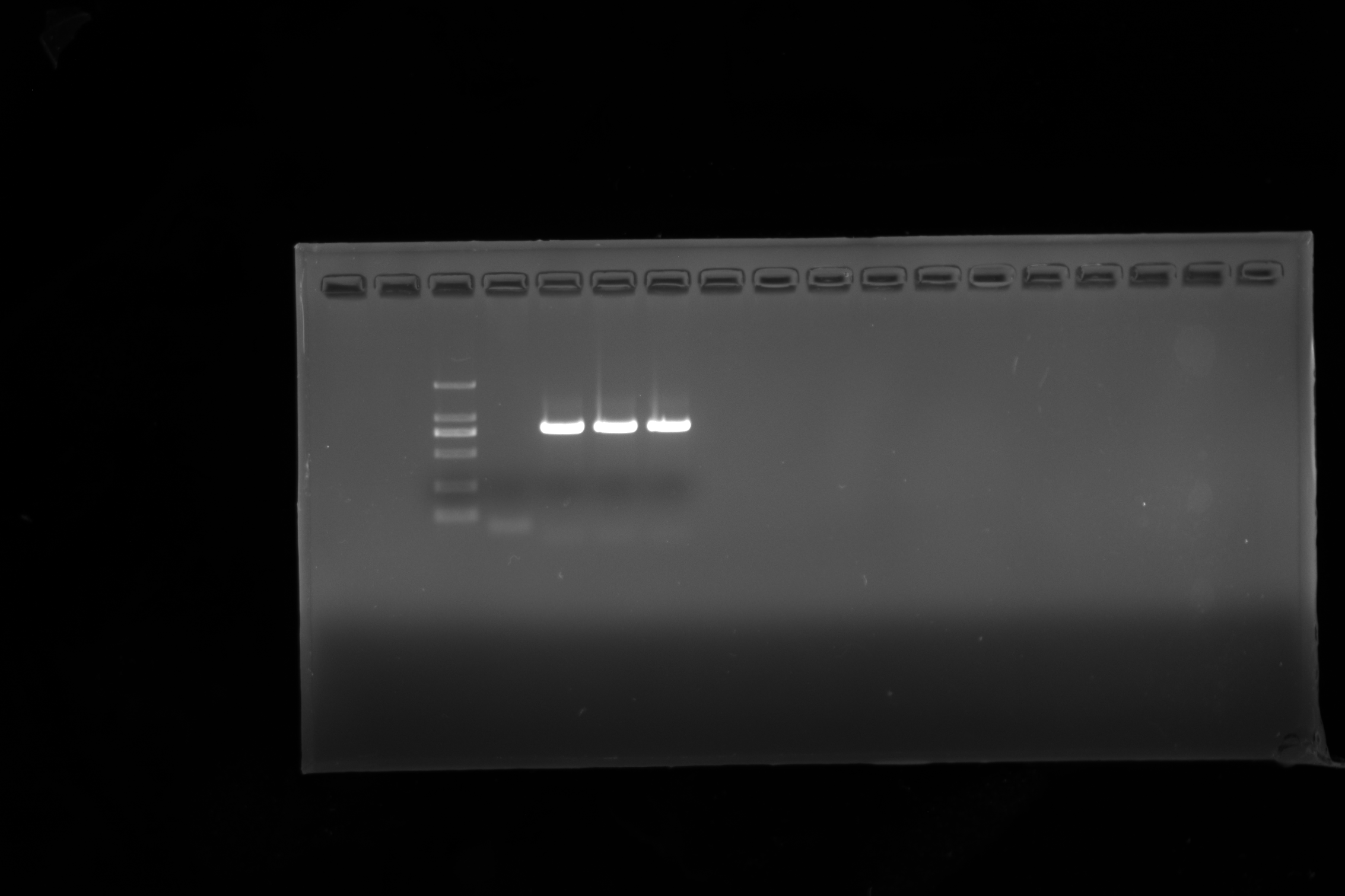

Supplement: Supplementary file 1 [file DataSheet1.zip › Supplementary Gel Images-1681421/FIG3E.tif]
